# Real-world effectiveness of pharmacological treatments for bipolar disorder: register-based national cohort study

**DOI:** 10.1192/bjp.2023.75

**Published:** 2023-10

**Authors:** Markku Lähteenvuo, Tapio Paljärvi, Antti Tanskanen, Heidi Taipale, Jari Tiihonen

**Affiliations:** Department of Forensic Psychiatry, University of Eastern Finland, Niuvanniemi Hospital, Kuopio, Finland; Department of Forensic Psychiatry, University of Eastern Finland, Niuvanniemi Hospital, Kuopio, Finland; and Department of Clinical Neuroscience, Division of Insurance Medicine, Karolinska Institutet, Stockholm, Sweden; Department of Forensic Psychiatry, University of Eastern Finland, Niuvanniemi Hospital, Kuopio, Finland; and Department of Clinical Neuroscience, Division of Insurance Medicine, Karolinska Institutet, Stockholm, Sweden; and School of Pharmacy, University of Eastern Finland, Kuopio, Finland; Department of Forensic Psychiatry, University of Eastern Finland, Niuvanniemi Hospital, Kuopio, Finland; Department of Clinical Neuroscience, Karolinska Institutet, Stockholm, Sweden; Centre for Psychiatry Research, Karolinska Institutet, Stockholm, Sweden; Stockholm Health Care Services, Region Stockholm, Stockholm, Sweden; and Neuroscience Center, University of Helsinki, Helsinki, Finland

**Keywords:** Bipolar affective disorders, epidemiology, pharmacotherapy, treatment effectiveness, lithium.

## Abstract

**Background:**

Pharmacological treatment patterns for bipolar disorder have changed during recent years, but for better or worse?

**Aims:**

To investigate the comparative real-world effectiveness of antipsychotics and mood stabilisers in bipolar disorder.

**Method:**

Register-based cohort study including all Finnish residents aged 16–65 with a diagnosis of bipolar disorder from in-patient care, specialised out-patient care, sickness absence and disability pensions registers between 1996 and 2018, with a mean follow-up of 9.3 years (s.d. = 6.4). Antipsychotic and mood stabiliser use was modelled using the PRE2DUP method and risk for hospital admission for psychiatric and non-psychiatric reasons when using versus not using medications was estimated using within-individual Cox models.

**Results:**

Among 60 045 individuals (56.4% female; mean age 41.7 years, s.d. = 15.8), the five medications associated with lowest risk of psychiatric admissions were olanzapine long-acting injection (LAI) (aHR = 0.54, 95% CI 0.37–0.80), haloperidol LAI (aHR = 0.62, 0.47–0.81), zuclopenthixol LAI (aHR = 0.66, 95% CI 0.52–0.85), lithium (aHR = 0.74, 95% CI 0.71–0.76) and clozapine (aHR = 0.75, 95% CI 0.64–0.87). Only ziprasidone (aHR = 1.26, 95% CI 1.07–1.49) was associated with a statistically higher risk. For non-psychiatric (somatic) admissions, only lithium (aHR = 0.77, 95% CI 0.74–0.81) and carbamazepine (aHR = 0.91, 95% CI 0.85–0.97) were associated with significantly reduced risk, whereas pregabalin, gabapentin and several oral antipsychotics, including quetiapine, were associated with an increased risk. Results for a subcohort of first-episode patients (26 395 individuals, 54.9% female; mean age 38.2 years, s.d. = 13.0) were in line with those of the total cohort.

**Conclusions:**

Lithium and certain LAI antipsychotics were associated with lowest risks of psychiatric admission. Lithium was the only treatment associated with decreased risk of both psychiatric and somatic admissions.

Bipolar disorder is a serious chronic mental disorder that affects >1% of the global population, with estimates of up to 4.4% in the USA.^1–3^ Bipolar disorder is divided into subtypes, with all subtypes displaying both depressive and manic symptoms to varying degrees and durations.^4^ Bipolar disorder carries a high risk of disability, need for hospital admission, and suicidality, with 30–50% of patients making suicide attempts.^5,6^ Bipolar disorder is also associated with markedly reduced life expectancy.^7^

## Treatment

Clinical care guidelines recommend the use of mood stabilisers, lithium first in line, and antipsychotics for the treatment of bipolar disorder.^8,9^ Antidepressants are sometimes recommended as adjunctive treatment, but results on the effectiveness of antidepressants are mixed,^10^ with concerns over their propensity to increase the risk of mania.^11^ Unfortunately, a recent study from the USA indicated that there is considerable heterogeneity in the care of bipolar disorder, and many people do not receive evidence-based care.^12^ As for the evidence base, meta-analyses of randomised controlled trials (RCTs) have shown mood stabilisers and second-generation antipsychotics to be roughly equal in effectiveness for bipolar disorder in general,^13^ although some treatments have seemed more appropriate in the manic, and others in the depressive, phase.^14–16^ However, since as many 90% of people with bipolar disorder have psychiatric comorbidities,^1^ conditions often leading to being excluded from RCTs, the results from RCTs may not reflect the effectiveness of these treatments in real-world settings. Indeed, recent observational studies have shown lithium to be more effective in the treatment of bipolar disorder than many second-generation antipsychotics or other mood stabilisers.^17,18^ In addition to effectiveness, different antipsychotics and mood stabilisers also have different propensities to cause side-effects, sometimes serious enough to lead to discontinuation or even warrant hospital admission.^15,19^ However, large-scale observational studies including a wide variety of different types of patient that have studied effectiveness and safety outcomes are still lacking.

## Present work

Here we present the largest observational cohort study to date not only exploring the comparative effectiveness and safety of antipsychotics and mood stabilisers in a nationwide cohort of people with bipolar disorder, but also looking specifically at first-episode patients.

## Method

### Study cohorts

The study population comprised all individuals between the ages of 16 and 65 years with a registered diagnosis of bipolar disorder (ICD-10: F30–F31) during the years 1987–2018 in any of the following Finnish national registers: the Care Register for Health Care (maintained by the National Institute of Health and Welfare, containing data on individuals treated in in-patient care and specialised out-patient care; data on in-patient care are available for the years 1987–2018 and out-patient care for 1998–2018), the sickness absence and disability pension registers (maintained by the Social Insurance Institution and the Finnish Centre for Pensions, containing also individuals without hospital or specialised out-patient care contact, if they had sick leave of over 14 days during the years 2004–2018 or were granted a disability pension owing to bipolar disorder during the years 1996–2018), after excluding all individuals with a schizophrenia-spectrum diagnosis (F20–F29) or dementia (F00–F03, G30) before their first bipolar disorder diagnosis. The main study cohort included 60 045 individuals. The cohort entry date for each individual was set at time of diagnosis of bipolar disorder or start of the observation period in 1996, whichever came later, and the cohort exit date was set at the end of the observation period at the end of 2018 or at death, whichever came sooner.

A subcohort of individuals with first-episode bipolar disorder was formed from the main study cohort. This was done by including persons having their first diagnosis during 1996–2018 and by excluding individuals who had used antipsychotic drugs (anatomical therapeutic chemical (ATC) code N05A excluding lithium) or mood stabilisers (N03AF01 carbamazepine, N03AG01 valproate, N03AX09 lamotrigine, N05AN01 lithium) within the 12 months before their first bipolar disorder diagnosis was recorded (26 395 were included in the incident cohort). This excluded a large number of participants, but ensured that the incident cohort population was without recent (1 year) exposure to the medications studied.

Each individual in Finland is assigned a unique personal identification number, which enabled us to link participants reliably between registers over the study period.

### Outcomes

The study cohorts were followed up for study outcomes during 1996–2018, the period for which we had information available on both study outcomes and exposures. Participants were additionally censored at death (*n* = 9666 died during follow-up). The main outcome in this study was psychiatric admission (defined as a hospital admission with an ICD-10 diagnosis of Fxx.xx). The secondary outcome measure was non-psychiatric admission (defined as a hospital admission with an ICD-10 diagnosis of anything other than Fxx.xx).

### Exposures

Mood stabilisers were defined as ATC codes N03AF01 carbamazepine, N03AG01 valproic acid, N03AX09 lamotrigine and N05AN01 lithium. Antipsychotics were defined as N05A excluding lithium, and were further categorised as either oral or long-acting injectable (LAI) formulations (oral formulation is meant if not specifically stated as LAI).

Drug use periods, referring to the time between when continuous medication use started and ended, were modelled using the PRE2DUP (‘from prescription drug purchases to drug use periods’) method, which is described in detail elsewhere.^20^ In short, the method is based on mathematical modelling of the sliding average of daily dose (in defined daily doses, DDDs). The modelling utilises prescription register data (obtained from the Social Insurance Institution and available for 1995–2018) on dispensing dates and amounts dispensed (but not prescribed doses, which are not stored) and expert-defined, drug package-specific parameters, which define upper and lower limits for daily dose. The method also takes into account personal regularity of use, stockpiling of drugs and days spent in hospital care. The time in hospital is omitted from the exposure periods, which is necessary because medications are provided by the caring unit during hospital admission and do not show up in the registries. The method has been validated in several ways.^21–23^ We used the PRE2DUP method to determine daily patterns of use and non-use for all the study medications. PRE2DUP tracks every medication individually and can therefore also be used to recognise periods of polypharmacy. The number, mean and median durations of drug use periods for the cohort are displayed in Supplementary Table 1, available at https://doi.org/10.1192/bjp.2023.75.

### Covariates

The analyses were adjusted for the use of antidepressants (N06A) and benzodiazepines and related drugs (N05BA, N05CD, N05CF). The within-individual models were additionally adjusted for temporal order of treatments, time since diagnosis of bipolar disorder and calendar age at the end of each interval. Covariates of between-individual models are described below, in Secondary analyses.

### Statistical analyses and covariates

#### Main analyses

Within-individual Cox regression analyses, described in detail in a previous publication^24^ and illustrated in Supplementary Fig. 1, were performed to calculate adjusted hazard ratios (aHR) for associations between periods of use/non-use of medications and the main and secondary outcomes (psychiatric and non-psychiatric admissions respectively). This was done in two separate sets: monotherapies with specific antipsychotics (when not more than one antipsychotic was used at a time) were compared with non-use of all antipsychotics (adjusted for concomitant mood stabiliser use) and monotherapies with specific mood stabilisers (when not more than one mood stabiliser was used at a time) were compared with non-use of all mood stabilisers (adjusted for antipsychotic use). Additionally, pooled variables ‘use of any oral antipsychotic’ and ‘use of any long-acting injectable antipsychotic’ were created by pooling usage data for oral antipsychotics together and long-acting injectables together and then comparing with non-use of antipsychotics. This was done also for mood stabilisers by pooling ‘use of any mood stabiliser’ and comparing it with non-use of mood stabilisers. Additional analyses comparing the use of antipsychotic polypharmacy (concurrent use of ≥2 antipsychotics) or mood stabiliser polypharmacy (concurrent use of ≥2 mood stabilisers) with not using that medication class (antipsychotics or mood stabilisers respectively) were performed as within-individual analyses for the whole cohort for the main outcome of psychiatric admission. The time reset used in the model was performed at the time of outcome. In addition to aHRs, 95% confidence intervals (95% CI) are reported. Exposures with fewer than 49 users and person-years of use are not reported. The within-individual analysis models were used to eliminate selection bias arising from permanent or semi-permanent individual characteristics such as genetics or long-term underlying severity of illness. The analyses were performed for both the full cohort and the incident cohort. An additional analysis comparing use of long-acting injectables with the use of their oral counterparts was performed for both outcomes. Finally, a head-to-head comparison comparing use of specific antipsychotic monotherapies with the most widely used antipsychotic (quetiapine) as well comparing specific mood stabiliser monotherapies with the most widely used mood stabiliser (valproic acid) were conducted for the main outcome of psychiatric admission for the whole cohort.

#### Secondary analyses

As secondary analyses, aHRs for the main outcome (psychiatric admission) were calculated using the traditional between-individuals Cox proportional hazards models, in which every individual in the study cohort contributed to the results. This analysis was adjusted for the following variables: gender, age at cohort entry, polypharmacy, order of dispensed medication, time since diagnosis of bipolar disorder, year of cohort entry and hospital admissions due to the following comorbidities: cancer, cardiovascular diseases, substance misuse, chronic obstructive pulmonary disease, diabetes, liver disease, renal disease, prior suicide attempt. The analysis was also stratified by the number of prior admissions for psychiatric reasons. To correct for protopathic bias an analysis omitting the 30 days of medication use from the beginning of each use period was carried out for both full cohort and incident cohort within-individual analyses. This was done to correct for bias arising from situations in which medication was initiated after worsening of symptoms, but did not take effect quickly enough to prevent an outcome. To account for the effect of mortality on the outcomes, analyses combining both main outcomes with deaths (psychiatric admission or death/non-psychiatric admission or death) were also conducted for the whole cohort.

The statistical analyses were performed between February 2022 and April 2023. Nominal *P*-values are shown throughout this paper. The main and secondary outcome analyses were corrected for multiple comparisons using the Benjamini–Hochberg false discovery rate method on a per graph basis. A corrected *P*-value of <0.05 was considered statistically significant.

### Ethical permissions

The research project was approved by the appropriate institutional authorities at the Finnish National Institute for Health and Welfare (permission 635/5.05.00/2019), the Social Insurance Institution of Finland (31/522/2019), Finnish Centre for Pensions (19023) and Statistics Finland (TK-53-569-19). Consent was not obtained from participants, as this is not required by Finnish law in registry studies where the privacy of the participants can be guaranteed.

## Results

### Cohort description

**Fig. 1:**
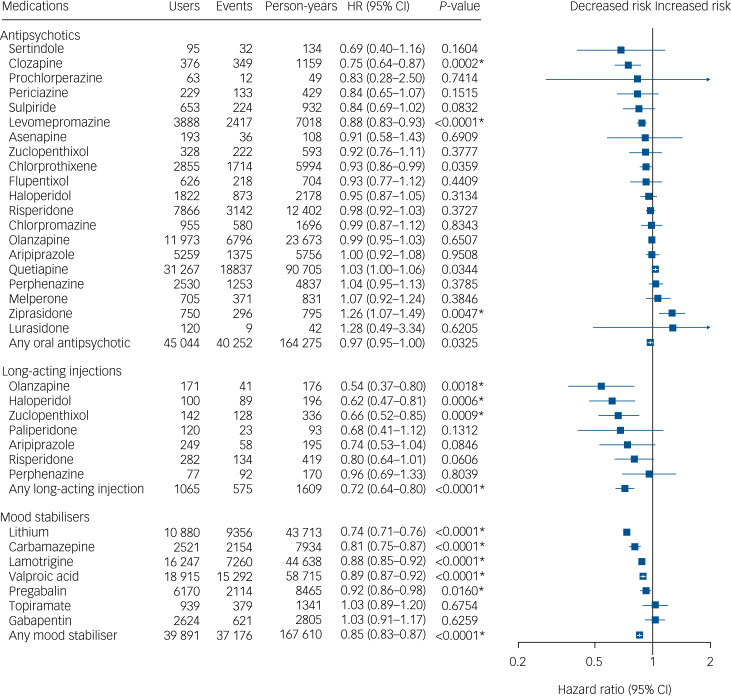
Risk of hospital admission for psychiatric reasons among individuals with bipolar disorder when using a medication versus not using a medication of the same medication class. Within-individual model based on 104 093 hospital admissions divided between 26 159 individuals. HR, adjusted hazard ratio. Nominal *P*-values are displayed. Results significant after correction for multiple comparisons using the Benjamini–Hochberg false discovery rate method at a 0.05 threshold are marked with an asterisk.

The full cohort included 60 045 individuals (56.4% female; mean age 41.7 years, s.d. = 15.8), with a mean follow-up time of 9.3 years (s.d. = 6.4). The incident cohort of newly diagnosed patients included 26 395 individuals (54.9% female; mean age 38.2 years, s.d. = 13.0), with a mean follow-up time of 9.2 years (s.d. = 5.6). The cohorts were quite similar regarding other recorded clinical characteristics. Characteristics for both cohorts are shown in Supplementary Table 2. The most commonly used antipsychotic was quetiapine (31 267 users), followed by olanzapine and risperidone, whereas the most common mood stabiliser was valproic acid (18 915 users), followed by lamotrigine and lithium (Fig. 1).

### Risk of psychiatric admission for the total cohort

A total of 104 093 psychiatric admission events were recorded during the follow-up, divided between 26 159 individuals. The results of within-individual analysis of the risk associations between using mood stabilisers or antipsychotics and being admitted to hospital for psychiatric reasons are presented in Fig. 1. The medications associated with a lower risk of psychiatric admissions were olanzapine LAI (aHR = 0.54, 95% CI 0.37–0.80), haloperidol LAI (aHR = 0.62, 95% CI 0.47–0.81), zuclopenthixol LAI (aHR = 0.66, 95% CI 0.52–0.85), lithium (aHR = 0.74, 95% CI 0.71–0.76), clozapine (aHR = 0.75, 95% CI 0.64–0.87), carbamazepine (aHR = 0.81, 95% CI 0.75–0.87), levomepromazine (aHR = 0.88, 95% CI 0.83–0.93), lamotrigine (aHR = 0.88, 95% CI 0.85–0.92), valproic acid (aHR = 0.89, 95% CI 0.87–0.92) and pregabalin (aHR = 0.92, 95% CI 0.86–0.98). Out of all the medications, only ziprasidone (aHR = 1.26, 95% CI 1.07–1.49) was associated with an increased risk of psychiatric admission. In general, use of long-acting injectables was associated with a 28% lower risk of psychiatric admission than non-use of antipsychotics (aHR = 0.72, 95% CI 0.64–0.80). The results for the long-acting injectable medications were very similar also when comparing use of the LAI and oral formulations (aHR = 0.72, 95% CI 0.64–0.81, data not displayed). Concurrent use of two or more antipsychotics (antipsychotic polypharmacy) was not associated with altered risk (aHR = 1.04, 95% CI 1.00–1.07) compared with non-use of antipsychotics. Concurrent use of two or more mood stabilisers (mood stabiliser polypharmacy) was associated with reduced risk of psychiatric admission (aHR = 0.86, 95% CI 0.83–0.89) compared with non-use of mood stabilisers. The data for polypharmacy as well as the most used polypharmacy combinations are shown in more detail in Supplementary Table 3. The results of head-to-head comparisons comparing the risk of psychiatric admission when using specific antipsychotic and mood stabiliser treatments with the most widely used counterpart (quetiapine for antipsychotics and valproic acid for mood stabilisers) are shown in Supplementary Table 4. The secondary analysis combining as an outcome psychiatric admission and death is shown in Supplementary Table 5. The results of this analysis were very similar to the main analysis.

The results of a secondary analysis correcting for protopathic bias by omitting the first 30 days of every new treatment showed similar results, although in that analysis paliperidone LAI (aHR = 0.32, 95% CI 0.15–0.67) was associated with decreased risk and topiramate (aHR = 1.22, 95% CI 1.04–1.43) with increased risk of psychiatric admission (Supplementary Figure 2). The between-individual analysis showed similar general trends as the within-individual analysis, although hazard ratios were markedly higher in the between-individuals analysis (Supplementary Table 6). Lamotrigine was the only treatment associated with a statistically significant reduced risk of psychiatric admission in the between-individual analysis (aHR = 0.86, 95% CI 0.83–0.89).

### Risk of psychiatric admission for the incident cohort

**Fig. 2:**
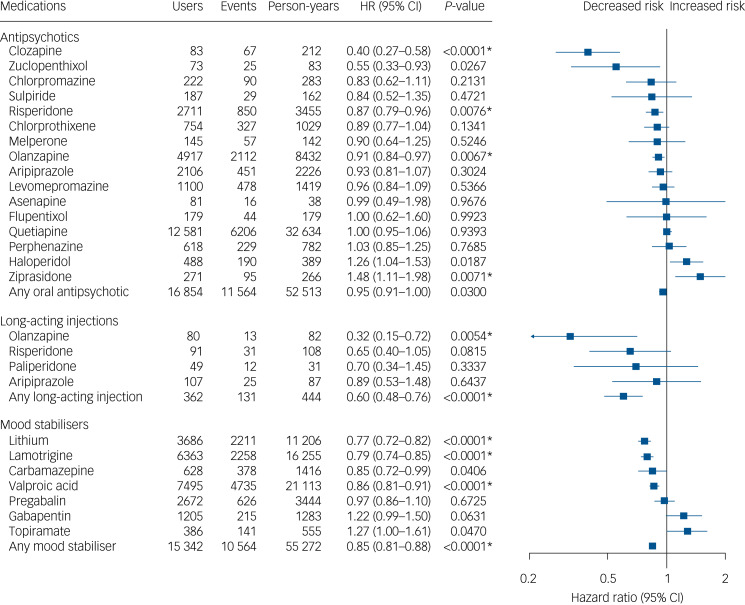
Risk of hospital admission for psychiatric reasons among individuals with incident bipolar disorder when using a medication versus not using a medication of the same medication class. Within-individual model based on 35 598 hospital admissions divided between 10 222 individuals. HR, adjusted hazard ratio. Nominal *P*-values are displayed. Results significant after correction for multiple comparisons using the Benjamini–Hochberg false discovery rate method at a 0.05 threshold are marked with an asterisk.

A total of 35 598 psychiatric admission events were recorded for the incident cohort during the follow-up, divided between 10 222 individuals. In the within-individual analysis of the incident cohort, olanzapine LAI (aHR = 0.32, 95% CI 0.15–0.72), clozapine (aHR = 0.40, 95% CI 0.27–0.58), lithium (aHR = 0.77, 95% CI 0.72–0.82), lamotrigine (aHR = 0.79, 95% CI 0.74–0.85), valproic acid (aHR = 0.86, 95% CI 0.81–0.91), risperidone (aHR = 0.87, 95% CI 0.79–0.96) and olanzapine (aHR = 0.91, 95% CI 0.84–0.97) were associated with decreased risk, and ziprasidone (aHR = 1.48, 95% CI 1.11–1.98) with increased risk of psychiatric admission (Fig. 2). Use of long-acting injectables in general was associated with a 40% reduced risk of psychiatric admission (aHR = 0.60, 95% CI 0.48–0.76) compared with non-use of antipsychotics in this cohort. The results of a secondary analysis correcting for protopathic bias by omitting the first 30 days of every new treatment in this cohort showed similar results, although in that analysis results for risperidone and oral olanzapine were not statistically significant, and topiramate and haloperidol were associated with an increased risk of psychiatric admission (Supplementary Figure 3).

### Risk of non-psychiatric admission for the total cohort

**Fig. 3:**
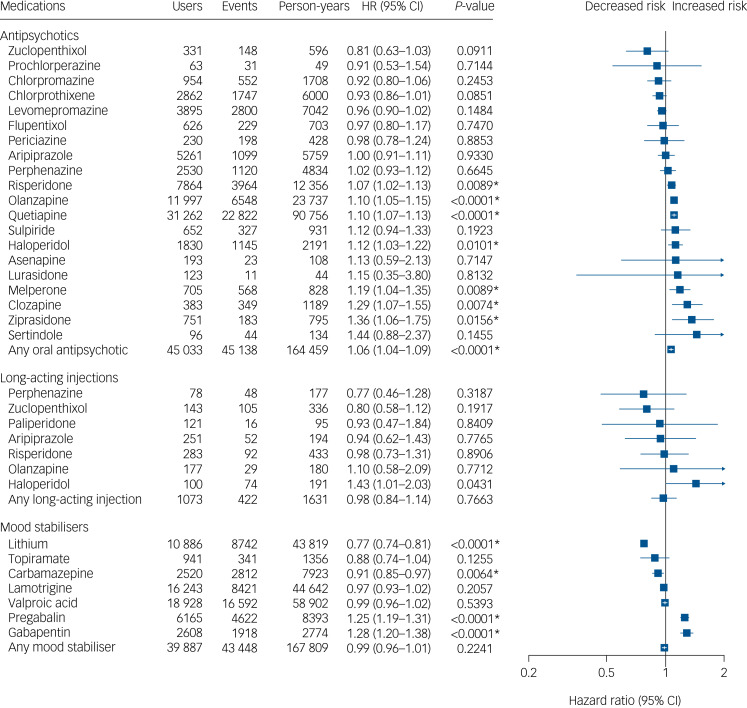
Risk of hospital admission for non-psychiatric reasons among individuals with bipolar disorder when using a medication versus not using a medication of the same medication class. Within-individual model based on 144 434 hospital admissions divided between 33 380 individuals. HR, adjusted hazard ratio. Nominal *P*-values are displayed. Results significant after correction for multiple comparisons using the Benjamini–Hochberg false discovery rate method at a 0.05 threshold are marked with an asterisk.

A total of 144 434 non-psychiatric (somatic) admission events were recorded during the follow-up, divided between 33 380 individuals. The results of the within-individual analysis of the risk associations between using individual mood stabilisers or antipsychotics and being admitted to hospital for non-psychiatric reasons are presented in Fig. 3. Of the studied medications, only lithium (aHR = 0.77, 95% CI 0.74–0.81) and carbamazepine (aHR = 0.91, 95% CI 0.85–0.97) were associated with significantly reduced risk of non-psychiatric admissions, whereas risperidone (aHR = 1.07, 95% CI 1.02–1.13), olanzapine (aHR = 1.10, 95% CI 1.05–1.15), quetiapine (aHR = 1.10, 95% CI 1.07–1.13), haloperidol (aHR = 1.12, 95% CI 1.03–1.22), melperone (aHR = 1.19, 95% CI 1.04–1.35), pregabalin (aHR = 1.25, 95% CI 1.19–1.31), gabapentin (aHR = 1.28, 95% CI 1.20–1.38), clozapine (aHR = 1.29, 95% CI 1.07–1.55) and ziprasidone (aHR = 1.36, 95% CI 1.06–1.75) were associated with significantly increased risk of non-psychiatric admission. In general, use of long-acting injectables was not associated with an altered risk for non-psychiatric admission compared with either using their oral counterparts (aHR = 0.91, 95% CI 0.78–1.06, data not displayed in figure) or non-use of antipsychotics (aHR = 0.98, 95% CI 0.84–1.14). The secondary analysis combining as an outcome non-psychiatric admission and death is shown in Supplementary Table 5. The results of this analysis were very similar to the main analysis. The results for a secondary analysis correcting for protopathic bias by omitting the first 30 days of every new treatment showed similar results, although carbamazepine was not associated with reduced risk and risperidone, haloperidol and clozapine, melperone and ziprasidone were not associated with increased risk (Supplementary Figure 4).

### Risk of non-psychiatric admission for the incident cohort

**Fig. 4:**
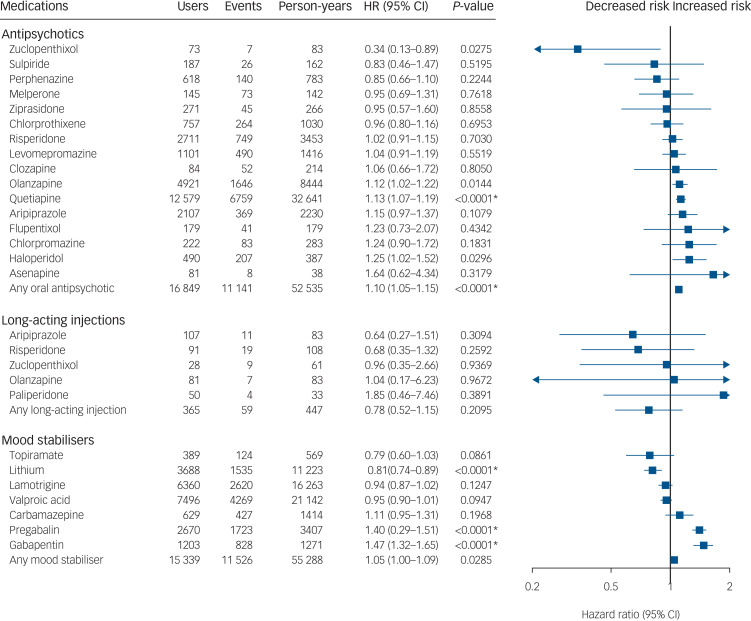
Risk of hospital admission for non-psychiatric reasons among individuals with incident bipolar disorder when using a medication versus not using a medication of the same medication class. Within-individual model based on 48 131 hospital admissions divided between 13 506 individuals. HR, adjusted hazard ratio. Nominal *P*-values are displayed. Results significant after correction for multiple comparisons using the Benjamini–Hochberg false discovery rate method at a 0.05 threshold are marked with an asterisk.

A total of 48 131 non-psychiatric (somatic) admission events were recorded for the incident cohort during the follow-up, divided between 13 506 individuals. In the within-individual analysis for the incident cohort, only lithium (aHR = 0.81, 95% CI 0.74–0.89) was associated with a reduced risk and quetiapine (1.13, 1.07–1.19), pregabalin (aHR = 1.40, 95% CI 1.29–1.51) and gabapentin (aHR = 1.47, 95% CI 1.32–1.65) were associated with increased risk of non-psychiatric admission (Fig. 4). The results of a secondary analysis correcting for protopathic bias by omitting the first 30 days of every new treatment in this cohort were the same as in the primary analysis (Supplementary Figure 5).

## Discussion

In this real-world observational national cohort study from Finland, we investigated for the first time the effectiveness of antipsychotics and mood stabilisers in preventing hospital admissions in bipolar disorder, both for in- and out-patients, but also looking specifically at first-episode patients. Our results are generally in line with other observational and randomised studies, as we also observed that using lithium, clozapine and long-acting injectable antipsychotics is associated with decreased risk of admission for psychiatric reasons in bipolar disorder.^19,25–29^ In this study, we present comparative real-world effectiveness with the largest cohort to date (more than three times larger than, for example, in our previous study^19^), the longest follow-up period and more representative sampling from nationwide registers covering also patients from non-specialised healthcare, which gave greater statistical power to look at incident patients and also allowed us to include in the analyses patients with less severe illness without hospital admissions. A striking finding in our study was that quetiapine, the most often used treatment in our study, was not associated with reduced risk of psychiatric admission, unlike several other treatments, but was associated with increased risk of non-psychiatric admission in both the total and incident cohorts, thus faring worse than several other treatments.

### Changes in treatment trends

Lithium has been the cornerstone of the treatment of bipolar disorder until recent years, but alarmingly there have been several reports that its use in bipolar disorder is declining.^30^ This is somewhat understandable, as modern second-generation antipsychotics such as quetiapine, which seem to have replaced lithium to a large extent, carry a lesser need for laboratory monitoring and dose titration.^30^ However, this trend of lithium use being replaced by use of quetiapine is alarming, as our results indicate that there are great differences in the risks of outcomes associated with the use of these two medications. Lithium was clearly associated with a lower risk of both psychiatric and non-psychiatric admissions, whereas quetiapine was not associated with reduced risk of psychiatric admission and was actually associated with an increased risk of non-psychiatric admission. Some studies, especially RCTs, have found lithium and quetiapine to have similar effectiveness in the general treatment of bipolar disorder,^31,32^ quetiapine in some studies outperforming lithium in the treatment of depression, but lithium having clear superiority in preventing self-harm. This discrepancy between RCTs and real-world studies may be because the RCTs exclude patients with comorbidities, and comorbidities are very prevalent in people with bipolar disorder and are likely to increase risks for negative outcomes, even fatal ones such as suicide. Clinicians also often have fears about the side-effect profile of lithium over other medications used to treat bipolar disorder, even though recent studies have shown that lithium has a lower side-effect burden than many other treatments.^31^ Thus, side-effects need to be balanced against the clinical benefit afforded by medications and the risks related to ineffective medications should be clearly communicated to patients.

### Ineffectiveness due to non-adherence

One primary reason for ineffectiveness is non-adherence.^27^ Non-adherence may also, to a certain extent, explain the better results seen for lithium than for quetiapine in our setting compared with RCTs, since RCTs measure symptoms during short-term follow-up in highly selected adherent patients. In real-life settings, patients may take their medications more irregularly unless they are monitored constantly, as happens during lithium treatment.^17^ In addition, long-acting injectables have also been offered as a solution to adherence problems.^27^ Long-acting injectables, of which for example olanzapine LAI was associated with the lowest risk of psychiatric admission in our study, could offer a strong alternative for patients not willing to try or with contraindications for lithium. Indeed, long-acting injectables in general were associated with a 28% lower risk of psychiatric admission in our study than their oral counterparts (i.e. the same pharmacological substance in different administration form), despite not being associated with an increased risk of non-psychiatric admission. However, the use of long-acting injectables remains low in healthcare settings despite the mounting evidence for their benefits in patients with bipolar disorder.^19,27,33^ Of note, the superiority of long-acting injectables was also evident in first-episode patients in our study.

### Strengths and weaknesses

As any other study, our study is not without limitations. The study population is inclusive and diverse on a national level, but being restricted to one country, may not be generalisable to populations of markedly different ethnicities or different healthcare systems. Also, all the results are based on data and end-points stored in national registries and do not come close to covering all of the clinical aspects and outcomes related to bipolar disorder or non-pharmacological treatments such as psychotherapy. We did not have data on the severity of symptoms or the quality of life of the patients included, and therefore cannot make inferences on the effects of medications on these important outcomes. Further, since suicidality in general is a frequent reason for admission to hospital, the results may be favouring compounds with more anti-suicidal properties. On the other hand, medications considered ‘more potent’ or with more side-effects might be reserved for more serious episodes and medications considered ‘less potent’ or with fewer side-effects used for milder episodes. Thus, the risk associations presented for medications considered clinically ‘more potent’, such as lithium, may underestimate the effectiveness of these medications. However, non-pharmacological treatments, such as psychotherapy or neuromodulation, may also be more frequently used for patients with more severe illness, which may inflate the effectiveness of the medications used for these individuals. The within-individual analyses used eliminate selection bias and bias arising from other long-term individual factors, but cannot correct for all sources of bias, such as protopathic bias, for which additional analyses were performed and were mostly in line with the primary results. Within-individual analyses also only include individuals with variation in both exposure (have changes in their medication) and outcome (be admitted to hospital), and are therefore somewhat exclusive. To correct for this, a between-individual analysis was performed for the main outcome in the total cohort, showing similar rank order as within-individual analysis. In general, hazard ratios were higher for all medications in the between-individual analysis. This was apparently related to low intrinsic risk among the most mildly ill patients with long periods of non-use of medications. There may also remain some skewing due to the effect of initial/previous outcome events on the risk of recurrence and to the effect of the length of previous hospital admissions on future treatment selection or course of disorder, for which the sensitivity analyses may not have been able to fully account.

### Implications

Our results challenge the widespread use of quetiapine in the treatment of bipolar disorder, since quetiapine was not associated with a reduced risk of psychiatric admission and was associated with an increased risk of non-psychiatric admission, and call for further research on the effectiveness and safety of quetiapine in the long-term and in real-world settings.

## Data Availability

The data used in this study can be requested from the registry holders and/or Findata following the appropriate protocols for registry data permissions.

## Supplementary material

For supplementary material accompanying this paper visit https://doi.org/10.1192/bjp.2023.75. click here to view supplementary material
