# Supplementary material for: Real-world effectiveness of pharmacological treatments for bipolar disorder: register-based national cohort study
Source: Br J Psychiatry. 2023 Oct;223(4):456–64. doi: 10.1192/bjp.2023.75 (PMC10866673; doi:10.1192/bjp.2023.75)
Supplement: Supplementary file 1 [file S0007125023000752sup.zip › S0007125023000752sup001.docx]

**Supplemental material for**

**Real-World Effectiveness of Pharmacological Treatments in Bipolar Disorder in a Finnish National Cohort of 60,045 Patients**

Markku Lähteenvuo, MD, PhD; Tapio Paljärvi, PhD; Antti Tanskanen, PhD; Heidi Taipale, PhD; Jari Tiihonen, MD, PhD

Department of Forensic Psychiatry, University of Eastern Finland, Niuvanniemi Hospital, Kuopio, Finland (All authors)

Department of Clinical Neuroscience, Division of Insurance Medicine, Karolinska Institutet, Stockholm, Sweden (Taipale, Tanskanen)

School of Pharmacy, University of Eastern Finland, Kuopio, Finland (Taipale)

Department of Clinical Neuroscience, Karolinska Institutet & Centre for Psychiatry Research, Stockholm Health Care Services, Region Stockholm, Stockholm, Sweden (Tiihonen)

Neuroscience Center, University of Helsinki, Helsinki, Finland (Tiihonen)

Corresponding author:

Markku Lähteenvuo, MD, PhD

Niuvanniemi Hospital

Niuvankuja 65, FI-70240 Kuopio, Finland

Tel. +358 295 242 347

[markku.lahteenvuo@niuva.fi](mailto:markku.lahteenvuo@niuva.fi)

| **Supplementary Table 1. Number (=amount), mean and median duration of antipsychotic (AP) and mood stabilizer (MS) use periods in the main analyses (days). LAI = long-acting injectable. SD = Standard Deviation.** | | | | | | |
| --- | --- | --- | --- | --- | --- | --- |
|  | N of use periods | Mean | SD | Median | Lower quartile | Upper quartile |
| ***Antipsychotics*** |  |  |  |  |  |  |
| Any antipsychotic | 467466 | 158.2 | 581.8 | 55 | 17 | 181 |
| AP non-use | 476702 | 247.5 | 711.1 | 75 | 22 | 243 |
| Quetiapine | 196875 | 184.6 | 601.5 | 65 | 20 | 205 |
| Olanzapine | 52941 | 146.5 | 588.6 | 57 | 20 | 187 |
| Risperidone | 27083 | 160.9 | 663.6 | 56 | 15 | 194 |
| Aripiprazole | 17068 | 155.6 | 504.5 | 61 | 21 | 191 |
| Levomepromazine | 16197 | 174.6 | 552.5 | 56 | 19 | 186 |
| Chlorprothixene | 11834 | 185.2 | 607.3 | 63 | 21 | 218 |
| Perphenazine | 10071 | 167.2 | 651.4 | 62 | 18 | 208 |
| Haloperidol | 5997 | 118.4 | 589.9 | 45 | 13 | 149 |
| Chlorpromazine | 3351 | 137.9 | 683.8 | 58 | 16 | 193 |
| Ziprasidone | 2333 | 142.3 | 467.7 | 50 | 16 | 153 |
| Melperone | 2297 | 113.7 | 634.6 | 47 | 15 | 157 |
| Sulpride | 2293 | 174.4 | 611.8 | 58 | 20 | 204 |
| Flupentixol | 1841 | 150.2 | 578.4 | 51 | 17 | 179 |
| Clozapine | 1750 | 223.3 | 664.3 | 80 | 22 | 259 |
| Zuclopenthixol | 1201 | 123.6 | 645.7 | 42 | 14 | 146 |
| Periciazine | 868 | 169.7 | 591.1 | 53 | 13 | 204.5 |
| Risperidone LAI | 830 | 142.2 | 602.1 | 64.5 | 15 | 193 |
| Aripiprazole LAI | 639 | 88.4 | 445.4 | 47 | 15 | 152 |
| Zuclopenthixol LAI | 629 | 126.8 | 707.4 | 67 | 17 | 218 |
| Asenapine | 459 | 72.1 | 459.1 | 30 | 15 | 105 |
| Haloperidol LAI | 366 | 157.6 | 668.9 | 54 | 14 | 195 |
| Paliperidone LAI | 342 | 123.6 | 406.9 | 21.5 | 8 | 139 |
| Olanzapine LAI | 333 | 84.2 | 652.8 | 65 | 13 | 217 |
| Sertindole | 330 | 163.0 | 455.9 | 69 | 18 | 171 |
| Perphenazine LAI | 295 | 148.8 | 824.6 | 50 | 8 | 227 |
| Lurasidone | 231 | 86.0 | 459.3 | 31 | 15 | 101 |
| Prochlorperazine | 176 | 166.9 | 360.7 | 35.5 | 29 | 160.5 |
| ***Mood stabilizers*** |  |  |  |  |  |  |
| Any mood stabilizer | 437185 | 167.2 | 557.5 | 57 | 20 | 182 |
| MS non-use | 506983 | 234.4 | 721.9 | 70 | 20 | 239 |
| Valproic acid | 126299 | 164.5 | 594.7 | 61 | 20 | 194 |
| Lamotrigine | 103847 | 171.9 | 524.4 | 64 | 22 | 194 |
| Lithium | 76384 | 215.9 | 685.6 | 69 | 21 | 228 |
| Pregabaline | 27280 | 122.6 | 392.9 | 43 | 16 | 130 |
| Carbamazine | 15413 | 182.5 | 631.1 | 57 | 20 | 198 |
| Gabapentin | 8991 | 127.1 | 371.2 | 49 | 20 | 128 |
| Topiramate | 3833 | 117.3 | 443.7 | 49 | 20 | 145 |

| **Supplementary Table 2. Characteristics of the study population (all) and incident cohort. SD =Standard Deviation** | | |
| --- | --- | --- |
|  | **All, N=60045** | **Incident, n=26395** |
| Mean age, years (SD) | 41.7 (15.8) | 38.2 (13.0) |
| Gender (%, n) |  |  |
| Male | 43.6 (26186) | 45.1 (11912) |
| Female | 56.4 (33859) | 54.9 (14483) |
| Calendar year of diagnosis (%, n) |  |  |
| <2000 | 14.1 (8452) | 7.7 (2024) |
| 2000-2009 | 44.5 (26744) | 50.7 (13377) |
| 2010-2018 | 41.4 (24849) | 41.7 (10994) |
| Previous hospitalization due to BD at baseline (%, n) | 24.3 (14600) | 23.3 (6160) |
| Psychiatric comorbidities (%, n) |  |  |
| Anxiety disorder | 26.6 (15965) | 25.5 (6733) |
| Personality disorder | 14.2 (8502) | 11.6 (3069) |
| Substance use disorder | 19.0 (11397) | 17.5 (4613) |
| Previous suicide attempt | 5.6 (3345) | 4.0 (1063) |

**Supplementary Figure 1. Description of the Within-Individual analysis used here.**

**
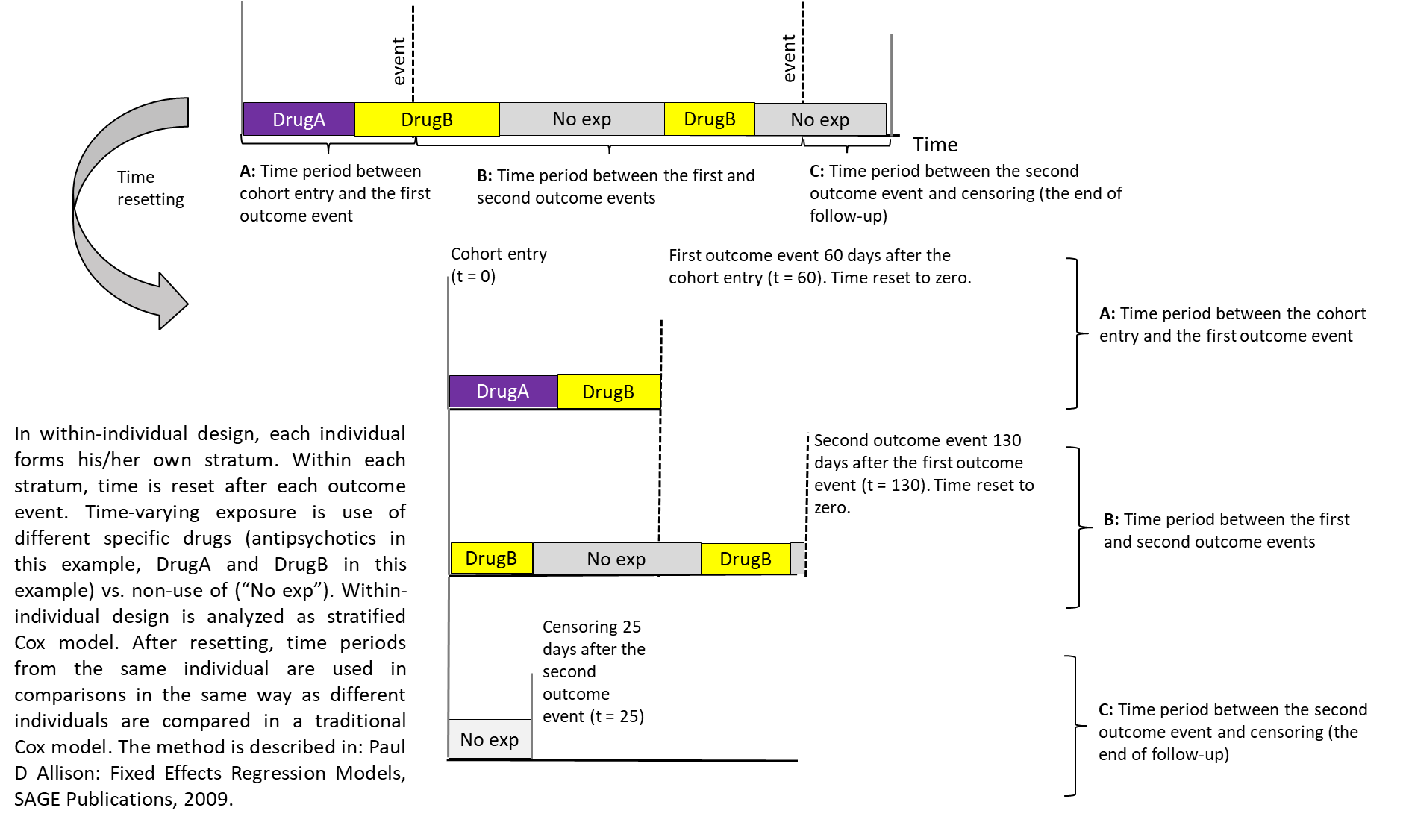
**

**
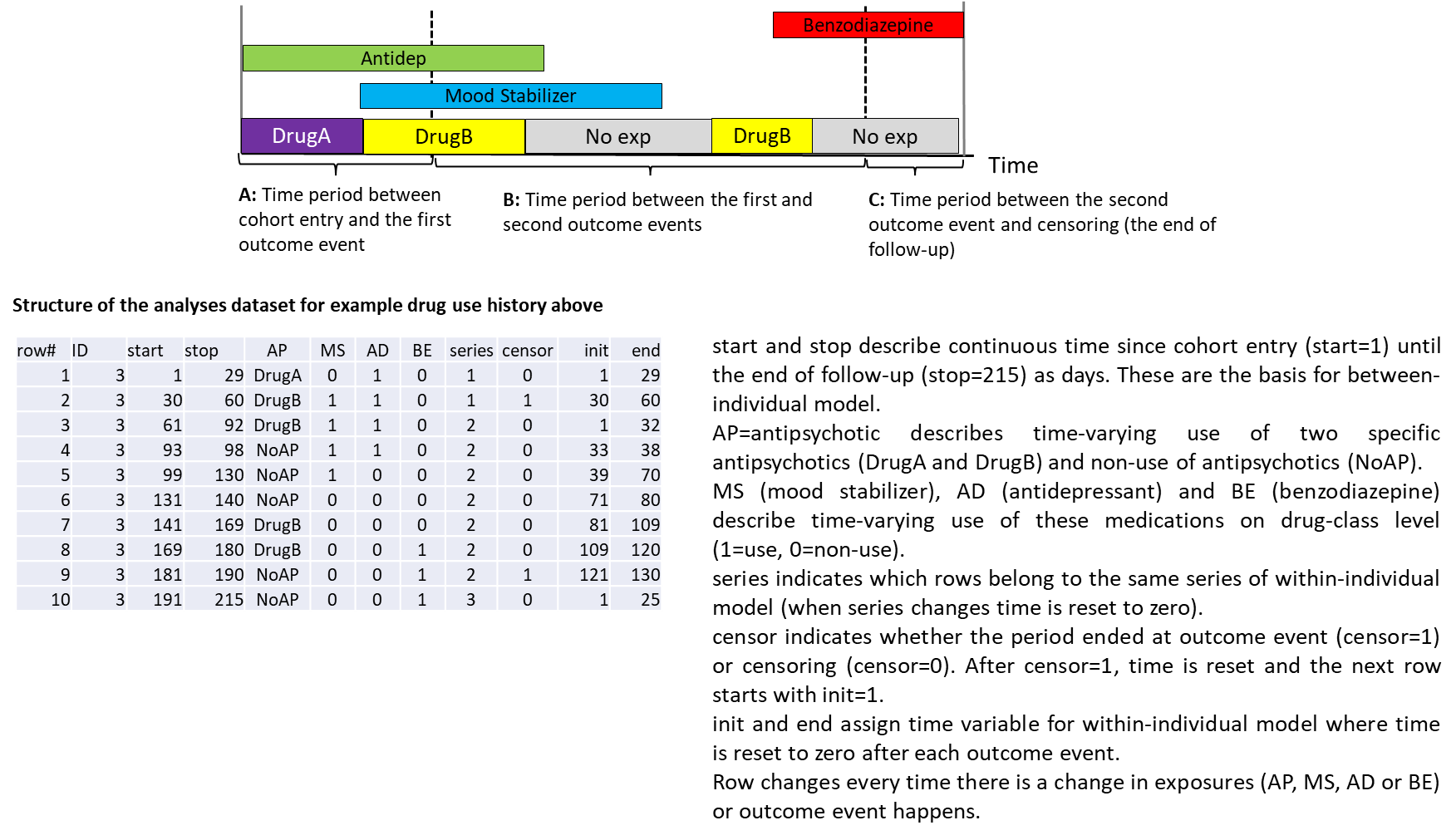
**

**Supplementary Figure 2. Within-individual risk of psychiatric hospitalization in the full cohort of individuals with bipolar disorder by medication compared to non-use of the same class of medication, with first 30 days of medication use omitted.** Nominal p-values are displayed. P-values significant after Benjamini-Hochberg False Discovery Rate correction for multiple comparisons at a 0.05 threshold are marked with an *. HR = adjusted Hazard Ratio. CI = Confidence Interval.

| **Supplementary Table 3. Within-individual risk of psychiatric hospitalization in the full cohort of individuals with bipolar disorder for polypharmacy of antipsychotics (≥2 antipsychotics concomitantly) and mood stabilizers (≥2 mood stabilizers concomitantly) compared to non-use of that medication class, including data on the number of use periods and duration of use for the most commonly used 2-drug combinations. IQR = Interquartile Range. CI = Confidence Interval.** | | |
| --- | --- | --- |
| *Antipsychotic polypharmacy* |  |  |
| Adjusted HR (95% CI) | 1.04 (1.00-1.07) |  |
| Events | 14517 |  |
| Users | 19958 |  |
| Person-years | 29565 |  |
| *Mood stabilizer polypharmacy* |  |  |
| Adjusted HR (95% CI) | 0.86 (0.83-0.89) |  |
| Events | 7632 |  |
| Users | 13265 |  |
| Person-years | 25205 |  |
|  |  |  |
| **Most common 2-drug combinations** | N of use periods | Median duration in days (IQR) |
| *Antipsychotic polypharmacy* |  |  |
| Olanzapine-Quetiapine | 11850 | 29 (14-82) |
| Quetiapine-Aripiprazole | 9701 | 35 (15-101) |
| Quetiapine-Risperidone | 5605 | 30 (14-90) |
| Olanzapine-Aripiprazole | 2532 | 29 (14-84) |
| Olanzapine-Risperidone | 1229 | 24 (12-57) |
| Risperidone-Aripiprazole | 487 | 25 (12-54) |
| *Mood stabilizer polypharmacy* |  |  |
| Valproic acid-Lamotrigine | 17045 | 54 (21-153) |
| Lamotrigine-Lithium | 13181 | 51 (19-148) |
| Valproic acid-Lithium | 10860 | 47 (18-126) |

| **Supplementary Table 4. Risk of psychiatric hospitalization among individuals with bipolar disorder in within-individual design with the most commonly used drug as a reference, namely Quetiapine for antipsychotics and Valproic acid for mood stabilizers. Adjusted Hazard Ratios (aHRs) with 95% Confidence Intervals (CI). LAI = long-acting injectable antipsychotic.** | |
| --- | --- |
| ***Antipsychotics, reference Quetiapine* aHR (95% CI)** | |
| Olanzapine LAI | 0.52 (0.36-0.77) |
| Haloperidol LAI | 0.60 (0.45-0.79) |
| Zuclopenthixol LAI | 0.64 (0.50-0.82) |
| Sertindole | 0.66 (0.39-1.13) |
| Paliperidone LAI | 0.66 (0.40-1.09) |
| Aripiprazole LAI | 0.72 (0.51-1.01) |
| Clozapine | 0.72 (0.62-0.84) |
| Risperidone LAI | 0.78 (0.62-0.98) |
| Prochlorperazine | 0.80 (0.27-2.42) |
| Periciazine | 0.81 (0.63-1.04) |
| Sulpride | 0.82 (0.67-0.99) |
| Levomepromazine | 0.85 (0.80-0.91) |
| Asenapine | 0.88 (0.56-1.39) |
| Zuclopenthixol | 0.89 (0.73-1.08) |
| Flupentixol | 0.90 (0.74-1.09) |
| Chlorprothixene | 0.90 (0.83-0.97) |
| Haloperidol | 0.92 (0.84-1.02) |
| Perphenazine LAI | 0.93 (0.67-1.29) |
| Risperidone | 0.95 (0.89-1.00) |
| Chlorpromazine | 0.96 (0.84-1.09) |
| Olanzapine | 0.96 (0.92-1.00) |
| Aripiprazole | 0.97 (0.89-1.05) |
| Perphenazine | 1.01 (0.92-1.10) |
| Melperone | 1.03 (0.89-1.20) |
| Ziprasidone | 1.22 (1.04-1.44) |
| Lurasidone | 1.24 (0.47-3.23) |
| ***Mood stabilizers, reference Valproic acid*** | |
| Lithium | 0.82 (0.79-0.86) |
| Carbamazine | 0.91 (0.84-0.97) |
| Lamotrigine | 0.99 (0.94-1.03) |
| Pregabaline | 1.03 (0.96-1.11) |
| Topiramate | 1.15 (0.99-1.33) |
| Gabapentin | 1.15 (1.01-1.31) |

| **Supplementary Table 5. Risk of psychiatric hospitalization or death and non-psychiatric hospitalization or death associated with use of specific antipsychotics (with non-use of antipsychotics as reference) and mood stabilizers (with non-use of moob stabilizers as reference). Adjusted Hazard Ratios (aHR) with 95% Confidence Intervals (CI). LAI = long-acting injectable antipsychotic.** | | | | | | | |
| --- | --- | --- | --- | --- | --- | --- | --- |
|  | |  |  | Psychiatric hospitalization | | Non-psychiatric hospitalization | |
|  | Users | | Person-years | Events | aHR (95%CI) | Events | aHR (95%CI) |
| *Antipsychotics* | |  |  |  |  |  |  |
| Antipsychotic non-use | | 53643 | 329187 | 42921 | reference | 70160 | reference |
| Chlorpromazine | | 955 | 1696 | 614 | 0.98 (0.86-1.11) | 581 | 0.92 (0.80-1.06) |
| Levomepromazine | | 3888 | 7018 | 2593 | 0.88 (0.83-0.94) | 2968 | 0.96 (0.90-1.02) |
| Dixyrazine | | 76 | 27 | 24 | 0.68 (0.43-1.08) | 20 | 1.03 (0.56-1.91) |
| Fluphenazine | | 43 | 333 | 7 | 0.61 (0.25-1.44) | 7 | 0.61 (0.23-1.61) |
| Perphenazine | | 2530 | 4837 | 1295 | 1.04 (0.96-1.13) | 1158 | 1.02 (0.93-1.12) |
| Perphenazine LAI | | 77 | 170 | 93 | 0.97 (0.70-1.33) | 49 | 0.76 (0.46-1.26) |
| Prochlorperazine | | 63 | 49 | 13 | 0.65 (0.23-1.88) | 31 | 0.88 (0.52-1.49) |
| Periciazine | | 229 | 429 | 138 | 0.81 (0.63-1.03) | 202 | 0.94 (0.75-1.19) |
| Haloperidol | | 1822 | 2178 | 952 | 0.94 (0.86-1.04) | 1215 | 1.11 (1.02-1.21) |
| Haloperidol LAI | | 100 | 196 | 96 | 0.62 (0.47-0.81) | 81 | 1.42 (1.00-2.00) |
| Melperone | | 705 | 831 | 400 | 0.98 (0.85-1.14) | 594 | 1.13 (1.00-1.28) |
| Molindone | | 6 | 3 | 3 | 0.58 (0.13-2.61) | 3 | 1.12 (0.09-14.33) |
| Sertindole | | 95 | 134 | 32 | 0.65 (0.39-1.10) | 44 | 1.38 (0.84-2.26) |
| Ziprasidone | | 750 | 795 | 302 | 1.24 (1.05-1.46) | 189 | 1.35 (1.05-1.73) |
| Lurasidone | | 120 | 42 | 9 | 1.22 (0.47-3.18) | 11 | 1.11 (0.34-3.66) |
| Flupentixol | | 626 | 704 | 228 | 0.93 (0.77-1.13) | 239 | 1.00 (0.83-1.21) |
| Flupentixol LAI | | 11 | 24 | 10 | 1.18 (0.53-2.66) | 6 | 0.41 (0.11-1.45) |
| Chlorprothixene | | 2855 | 5994 | 1812 | 0.92 (0.86-0.99) | 1834 | 0.93 (0.86-1.01) |
| Zuclopenthixol | | 328 | 593 | 237 | 0.91 (0.75-1.10) | 161 | 0.82 (0.64-1.04) |
| Zuclopenthixol LAI | | 142 | 336 | 136 | 0.65 (0.51-0.83) | 113 | 0.79 (0.57-1.09) |
| Clozapine | | 376 | 1159 | 364 | 0.73 (0.63-0.85) | 362 | 1.29 (1.07-1.55) |
| Olanzapine | | 11973 | 23673 | 7218 | 0.99 (0.95-1.03) | 6920 | 1.10 (1.05-1.15) |
| Olanzapine LAI | | 171 | 176 | 45 | 0.57 (0.40-0.83) | 34 | 1.10 (0.59-2.03) |
| Quetiapine | | 31267 | 90705 | 19778 | 1.02 (0.99-1.06) | 23660 | 1.09 (1.06-1.12) |
| Asenapine | | 193 | 108 | 39 | 0.93 (0.59-1.45) | 26 | 1.26 (0.68-2.33) |
| Sulpride | | 653 | 932 | 239 | 0.83 (0.68-1.00) | 337 | 1.08 (0.91-1.27) |
| Risperidone | | 7866 | 12402 | 3384 | 0.98 (0.92-1.03) | 4175 | 1.06 (1.01-1.12) |
| Risperidone LAI | | 282 | 419 | 142 | 0.81 (0.65-1.02) | 99 | 0.97 (0.73-1.29) |
| Aripiprazole | | 5259 | 5756 | 1412 | 0.99 (0.91-1.07) | 1133 | 1.00 (0.91-1.11) |
| Aripiprazole LAI | | 249 | 195 | 61 | 0.76 (0.54-1.06) | 55 | 1.01 (0.68-1.51) |
| Paliperidone LAI | | 120 | 93 | 23 | 0.65 (0.40-1.08) | 16 | 0.92 (0.46-1.81) |
| Antipsychotic polytherapy | | 19958 | 29565 | 15051 | 1.03 (1.00-1.06) | 10386 | 1.08 (1.04-1.12) |
| *Mood stabilizers* | |  |  |  |  |  |  |
| Mood stabilizer non-use | | 54685 | 330205 | 53498 | reference | 74461 | reference |
| Carbamazine | | 2521 | 7934 | 2301 | 0.80 (0.75-0.86) | 2933 | 0.90 (0.84-0.96) |
| Valproic acid | | 18915 | 58718 | 16079 | 0.89 (0.86-0.92) | 17283 | 0.98 (0.95-1.02) |
| Lamotrigine | | 16247 | 44640 | 7578 | 0.88 (0.84-0.92) | 8694 | 0.97 (0.93-1.01) |
| Topiramate | | 939 | 1341 | 385 | 1.01 (0.87-1.17) | 347 | 0.88 (0.74-1.04) |
| Gabapentin | | 2624 | 2804 | 679 | 1.04 (0.91-1.18) | 1970 | 1.27 (1.19-1.37) |
| Pregabaline | | 6170 | 8465 | 2331 | 0.93 (0.87-0.99) | 4812 | 1.26 (1.20-1.32) |
| Lithium | | 10880 | 43714 | 9695 | 0.72 (0.69-0.74) | 9053 | 0.76 (0.73-0.79) |
| Mood Stabilizer polytherapy | | 13265 | 25205 | 7912 | 0.85 (0.82-0.88) | 8120 | 1.08 (1.03-1.12) |

**Supplementary Table 6. Between-individual risk of psychiatric hospitalization in the full cohort of individuals with bipolar disorder by medication compared to non-use of the same class of medication. Nominal p-values are displayed. HR = adjusted Hazard Ratio. CI = Confidence Interval.**

|  | **Hospitalisations due to mental health outcomes** | | |
| --- | --- | --- | --- |
| **Antipsychotics** | **HR** | **95% CI** | **P-value** |
| Reference (non-use) | 1.00 | reference |  |
| olanzapine LAI | 0.85 | 0.58, 1.27 | 0.437 |
| paliperidone LAI | 1.00 | 0.59, 1.69 | 0.993 |
| clozapine | 1.03 | 0.82, 1.30 | 0.793 |
| sertindole | 1.06 | 0.74, 1.51 | 0.769 |
| aripiprazole LAI | 1.10 | 0.81, 1.49 | 0.536 |
| fluphenazine | 1.11 | 0.48, 2.58 | 0.804 |
| lurasidone | 1.18 | 0.61, 2.28 | 0.634 |
| quetiapine | 1.18 | 1.14, 1.21 | <.001 |
| periciazine | 1.19 | 0.88, 1.60 | 0.251 |
| zuclopenthixol LAI | 1.19 | 0.84, 1.69 | 0.332 |
| sulpiride | 1.24 | 1.05, 1.47 | 0.013 |
| levomepromazine | 1.26 | 1.18, 1.35 | <.001 |
| risperidone | 1.26 | 1.19, 1.34 | <.001 |
| chlorprothixene | 1.31 | 1.21, 1.42 | <.001 |
| risperidone LAI | 1.32 | 1.05, 1.68 | 0.020 |
| thioridazine | 1.33 | 1.20, 1.48 | <.001 |
| olanzapine | 1.33 | 1.27, 1.39 | <.001 |
| aripiprazole | 1.33 | 1.23, 1.45 | <.001 |
| dixyrazine | 1.38 | 0.75, 2.52 | 0.302 |
| flupentixol | 1.44 | 1.23, 1.68 | <.001 |
| chlorpromazine | 1.46 | 1.28, 1.67 | <.001 |
| haloperidol LAI | 1.49 | 1.08, 2.07 | 0.016 |
| zuclopenthixol | 1.49 | 1.12, 1.99 | 0.007 |
| perphenazine | 1.50 | 1.39, 1.62 | <.001 |
| haloperidol | 1.52 | 1.38, 1.68 | <.001 |
| melperone | 1.54 | 1.27, 1.88 | <.001 |
| prochlorperazine | 1.62 | 0.78, 3.37 | 0.194 |
| asenapine | 1.68 | 1.20, 2.35 | 0.003 |
| ziprasidone | 1.75 | 1.48, 2.08 | <.001 |
| flupentixol LAI | 1.75 | 0.97, 3.17 | 0.064 |
| perphenazine LAI | 2.28 | 1.28, 4.05 | 0.005 |
| molindone | 3.26 | 0.89, 11.99 | 0.075 |
| Mood stabilisers |  |  |  |
| Reference (non-use) | 1.00 | reference |  |
| lamotrigine | 0.86 | 0.83, 0.89 | <.001 |
| lithium | 1.02 | 0.99, 1.06 | 0.221 |
| valproic acid | 1.05 | 1.02, 1.08 | 0.002 |
| pregabalin | 1.05 | 0.98, 1.12 | 0.192 |
| carbamazepine | 1.06 | 0.98, 1.14 | 0.171 |
| topiramate | 1.08 | 0.91, 1.29 | 0.366 |
| gabapentin | 1.18 | 1.04, 1.34 | 0.010 |

**Supplementary Figure 3. Within-individual risk of psychiatric hospitalization in the incident cohort of individuals with bipolar disorder by medication compared to non-use of the same class of medication, with first 30 days of medication use omitted.** Nominal p-values are displayed. P-values significant after Benjamini-Hochberg False Discovery Rate correction for multiple comparisons at a 0.05 threshold are marked with an *. HR = adjusted Hazard Ratio. CI = Confidence Interval.

**Supplementary Figure 4. Within-individual risk of non-psychiatric hospitalization in the total cohort of individuals with bipolar disorder by medication compared to non-use of the same class of medication, with first 30 days of medication use omitted.** Nominal p-values are displayed. P-values significant after Benjamini-Hochberg False Discovery Rate correction for multiple comparisons at a 0.05 threshold are marked with an *. HR = adjusted Hazard Ratio. CI = Confidence Interval.

**Supplementary Figure 5. Within-individual risk of non-psychiatric hospitalization in the incident cohort of individuals with bipolar disorder by medication compared to non-use of the same class of medication, with first 30 days of medication use omitted.** Nominal p-values are displayed. P-values significant after Benjamini-Hochberg False Discovery Rate correction for multiple comparisons at a 0.05 threshold are marked with an *. HR = adjusted Hazard Ratio. CI = Confidence Interval.
