# Supplementary material for: Real-world effectiveness of pharmacological treatments for bipolar disorder: register-based national cohort study
Source: Br J Psychiatry. 2023 Oct;223(4):456–64. doi: 10.1192/bjp.2023.75 (PMC10866673; doi:10.1192/bjp.2023.75)
Supplement: Supplementary file 1 [file S0007125023000752sup.zip › S0007125023000855sup002.doc]

sTable 1. Relative Risk of schizophrenia between FDRs of individuals who committed suicide and matched cohort, stratified by kinships

| Type of Affected Relative | Sex of relative | Sex of individual | Number of Schizophrenia (%) | | Adjusted RRǂ  (95C.I.) |
| --- | --- | --- | --- | --- | --- |
|  |  |  | FDRs of CS individuals | Matched Cohort |  |
| Any | Female | Female | 125 (1.25) | 233 (0.58) | 1.92* (1.55, 2.39) |
|  |  | Male | 135 (1.17) | 292 (0.63) | 1.67* (1.36, 2.06) |
|  |  | All | 260 (1.21) | 525 (0.61) | 1.79* (1.54, 2.08) |
|  | Male | Female | 225 (1.00) | 531 (0.59) | 1.49* (1.27, 1.75) |
|  |  | Male | 261 (1.08) | 681 (0.70) | 1.34* (1.16, 1.54) |
|  |  | All | 486 (1.04) | 1212 (0.65) | 1.40* (1.26, 1.56) |
|  | All | Female | 350 (1.08) | 764 (0.59) | 1.63* (1.43, 1.85) |
|  |  | Male | 396 (1.11) | 973 (0.68) | 1.45* (1.28, 1.63) |
|  |  | All | 746 (1.09) | 1737 (0.64) | 1.53* (1.40, 1.66) |
| Parents | Female  (mother) | Female | 49 (0.93) | 118 (0.56) | 1.48 (1.06, 2.06) |
|  |  | Male | 81 (1.20) | 187 (0.69) | 1.64* (1.26, 2.14) |
|  |  | All | 130 (1.08) | 305 (0.64) | 1.57* (1.28, 1.94) |
|  | Male  (father) | Female | 96 (0.81) | 264 (0.56) | 1.30 (1.03, 1.65) |
|  |  | Male | 126 (0.89) | 402 (0.71) | 1.12 (0.92, 1.37) |
|  |  | All | 222 (0.85) | 666 (0.64) | 1.19 (1.02, 1.39) |
|  | All | Female | 145 (0.85) | 382 (0.56) | 1.36 (1.12, 1.65) |
|  |  | Male | 207 (0.99) | 589 (0.70) | 1.28 (1.09, 1.50) |
|  |  | All | 352 (0.93) | 971 (0.64) | 1.31* (1.16, 1.48) |
| Offspring | Female  (daughter) | Female | 33 (1.41) | 52 (0.56) | 2.31* (1.50, 3.56) |
|  |  | Male | 13 (0.61) | 35 (0.41) | 1.37 (0.71, 2.64) |
|  |  | All | 46 (1.03) | 87 (0.49) | 1.93* (1.34, 2.76) |
|  | Male  (son) | Female | 73 (1.28) | 112 (0.49) | 2.33* (1.71, 3.17) |
|  |  | Male | 36 (0.72) | 80 (0.40) | 1.63 (1.09, 2.44) |
|  |  | All | 109 (1.02) | 192 (0.45) | 2.04* (1.60, 2.60) |
|  | All | Female | 106 (1.32) | 164 (0.51) | 2.32* (1.81, 2.98) |
|  |  | Male | 49 (0.69) | 115 (0.40) | 1.55 (1.10, 2.18) |
|  |  | All | 155 (1.02) | 279 (0.46) | 2.00* (1.64, 2.45) |
| Siblings | Female  (Sister) | Female | 43 (1.76) | 63 (0.64) | 2.62* (1.76, 3.90) |
|  |  | Male | 41 (1.56) | 70 (0.67) | 2.09* (1.41, 3.11) |
|  |  | All | 84 (1.66) | 133 (0.66) | 2.34* (1.77, 3.10) |
|  | Male  (brother) | Female | 56 (1.15) | 155 (0.79) | 1.29 (0.95, 1.76) |
|  |  | Male | 99 (1.96) | 199 (0.98) | 1.73* (1.35, 2.21) |
|  |  | All | 155 (1.56) | 354 (0.89) | 1.54* (1.27, 1.87) |
|  | All | Female | 99 (1.35) | 218 (0.74) | 1.66* (1.30, 2.11) |
|  |  | Male | 140 (1.82) | 269 (0.88) | 1.82* (1.47, 2.25) |
|  |  | All | 239 (1.59) | 487 (0.81) | 1.75* (1.49, 2.05) |

RR, relative risk

* indicates the statistical significance after the Bonferroni correction.

sTable 2. Relative Risk of Bipolar disorder between FDRs of individuals who committed suicide and matched cohort, stratified by kinships

| Type of Affected Relative | Sex of relative | Sex of individual | Number of Bipolar disorder (%) | | Adjusted RRǂ  (95C.I.) |
| --- | --- | --- | --- | --- | --- |
|  |  |  | FDRs of CS individuals | Matched Cohort |  |
| Any | Female | Female | 219 (2.18) | 273 (0.68) | 3.08* (2.59, 3.67) |
|  |  | Male | 163 (1.42) | 261 (0.57) | 2.40* (1.97, 2.92) |
|  |  | All | 382 (1.77) | 534 (0.62) | 2.75* (2.41, 3.13) |
|  | Male | Female | 303 (1.35) | 631 (0.70) | 1.78* (1.55, 2.04) |
|  |  | Male | 224 (0.93) | 559 (0.58) | 1.51* (1.29, 1.77) |
|  |  | All | 527 (1.13) | 1190 (0.64) | 1.65* (1.49, 1.84) |
|  | All | Female | 522 (1.61) | 904 (0.70) | 2.17* (1.94, 2.41) |
|  |  | Male | 387 (1.08) | 820 (0.57) | 1.79* (1.59, 2.03) |
|  |  | All | 909 (1.33) | 1724 (0.63) | 1.99* (1.83, 2.16) |
| Parents | Female  (mother) | Female | 91 (1.73) | 125 (0.59) | 2.78* (2.12, 3.63) |
|  |  | Male | 82 (1.22) | 163 (0.61) | 1.95* (1.49, 2.54) |
|  |  | All | 173 (1.44) | 288 (0.60) | 2.31* (1.91, 2.78) |
|  | Male  (father) | Female | 123 (1.03) | 286 (0.60) | 1.57* (1.27, 1.95) |
|  |  | Male | 106 (0.75) | 298 (0.53) | 1.34 (1.07, 1.68) |
|  |  | All | 229 (0.88) | 584 (0.56) | 1.46* (1.25, 1.70) |
|  | All | Female | 214 (1.25) | 411 (0.60) | 1.93* (1.64, 2.28) |
|  |  | Male | 188 (0.90) | 461 (0.55) | 1.55* (1.31, 1.84) |
|  |  | All | 402 (1.06) | 872 (0.57) | 1.73* (1.54, 1.95) |
| Offspring | Female  (daughter) | Female | 68 (2.91) | 65 (0.70) | 3.95* (2.85, 5.49) |
|  |  | Male | 32 (1.49) | 44 (0.51) | 2.78* (1.73, 4.49) |
|  |  | All | 100 (2.23) | 109 (0.61) | 3.50* (2.67, 4.59) |
|  | Male  (son) | Female | 93 (1.64) | 163 (0.72) | 2.18* (1.68, 2.83) |
|  |  | Male | 51 (1.02) | 114 (0.57) | 1.74* (1.25, 2.43) |
|  |  | All | 144 (1.35) | 277 (0.65) | 2.00* (1.63, 2.46) |
|  | All | Female | 161 (2.01) | 228 (0.71) | 2.70* (2.20, 3.30) |
|  |  | Male | 83 (1.16) | 158 (0.55) | 2.04* (1.55, 2.67) |
|  |  | All | 244 (1.61) | 386 (0.64) | 2.43* (2.07, 2.85) |
| Siblings | Female  (Sister) | Female | 60 (2.46) | 83 (0.85) | 2.87* (2.06, 4.00) |
|  |  | Male | 49 (1.86) | 54 (0.51) | 3.45* (2.34, 5.11) |
|  |  | All | 109 (2.15) | 137 (0.68) | 3.11* (2.42, 4.00) |
|  | Male  (brother) | Female | 87 (1.78) | 182 (0.93) | 1.77* (1.37, 2.28) |
|  |  | Male | 67 (1.33) | 147 (0.73) | 1.68* (1.24, 2.26) |
|  |  | All | 154 (1.55) | 329 (0.83) | 1.73* (1.42, 2.10) |
|  | All | Female | 147 (2.01) | 265 (0.90) | 2.11* (1.72, 2.57) |
|  |  | Male | 116 (1.51) | 201 (0.65) | 2.14* (1.69, 2.70) |
|  |  | All | 263 (1.75) | 466 (0.78) | 2.12* (1.82, 2.47) |

RR, relative risk

* indicates the statistical significance after the Bonferroni correction.

sTable 3. Relative Risk of MDD between FDRs of individuals who committed suicide and matched cohort, stratified by kinships

| Type of Affected Relative | Sex of relative | Sex of individual | Number of MDD (%) | | Adjusted RRǂ  (95C.I.) |
| --- | --- | --- | --- | --- | --- |
|  |  |  | FDRs of CS individuals | Matched Cohort |  |
| Any | Female | Female | 602 (6.00) | 956 (2.38) | 2.51* (2.26, 2.79) |
|  |  | Male | 401 (3.49) | 728 (1.58) | 2.19* (1.93, 2.48) |
|  |  | All | 1003 (4.66) | 1684 (1.95) | 2.37* (2.19, 2.57) |
|  | Male | Female | 955 (4.25) | 2038 (2.27) | 1.87* (1.73, 2.02) |
|  |  | Male | 613 (2.53) | 1425 (1.47) | 1.70* (1.54, 1.87) |
|  |  | All | 1568 (3.36) | 3463 (1.86) | 1.80* (1.69, 1.91) |
|  | All | Female | 1557 (4.79) | 2994 (2.30) | 2.07* (1.94, 2.21) |
|  |  | Male | 1014 (2.84) | 2153 (1.51) | 1.86* (1.73, 2.01) |
|  |  | All | 2571 (3.77) | 5147 (1.89) | 1.98* (1.89, 2.08) |
| Parents | Female  (mother) | Female | 239 (4.54) | 403 (1.91) | 2.34* (1.98, 2.75) |
|  |  | Male | 209 (3.11) | 401 (1.49) | 2.05* (1.73, 2.44) |
|  |  | All | 448 (3.74) | 804 (1.68) | 2.20* (1.95, 2.47) |
|  | Male  (father) | Female | 345 (2.90) | 796 (1.67) | 1.69* (1.49, 1.93) |
|  |  | Male | 282 (1.99) | 715 (1.26) | 1.54* (1.34, 1.78) |
|  |  | All | 627 (2.41) | 1511 (1.45) | 1.62* (1.48, 1.79) |
|  | All | Female | 584 (3.41) | 1199 (1.75) | 1.91* (1.72, 2.11) |
|  |  | Male | 491 (2.35) | 1116 (1.34) | 1.73* (1.55, 1.92) |
|  |  | All | 1075 (2.83) | 2315 (1.52) | 1.82* (1.69, 1.96) |
| Offspring | Female  (daughter) | Female | 226 (9.68) | 337 (3.61) | 2.70* (2.27, 3.23) |
|  |  | Male | 98 (4.57) | 168 (1.96) | 2.34* (1.80, 3.04) |
|  |  | All | 324 (7.23) | 505 (2.82) | 2.58* (2.23, 2.98) |
|  | Male  (son) | Female | 379 (6.67) | 755 (3.32) | 2.11* (1.86, 2.40) |
|  |  | Male | 158 (3.17) | 384 (1.92) | 1.64* (1.35, 1.99) |
|  |  | All | 537 (5.03) | 1139 (2.67) | 1.94* (1.74, 2.16) |
|  | All | Female | 605 (7.54) | 1092 (3.40) | 2.29* (2.07, 2.54) |
|  |  | Male | 256 (3.59) | 552 (1.93) | 1.85* (1.59, 2.16) |
|  |  | All | 861 (5.68) | 1644 (2.71) | 2.14* (1.96, 2.33) |
| Siblings | Female  (Sister) | Female | 137 (5.61) | 216 (2.21) | 2.57* (2.05, 3.21) |
|  |  | Male | 94 (3.58) | 159 (1.51) | 2.37* (1.83, 3.08) |
|  |  | All | 231 (4.56) | 375 (1.85) | 2.48* (2.09, 2.94) |
|  | Male  (brother) | Female | 231 (4.73) | 487 (2.49) | 1.84* (1.56, 2.16) |
|  |  | Male | 173 (3.42) | 326 (1.61) | 2.06* (1.71, 2.49) |
|  |  | All | 404 (4.06) | 813 (2.04) | 1.92* (1.70, 2.17) |
|  | All | Female | 368 (5.02) | 703 (2.40) | 2.06* (1.81, 2.35) |
|  |  | Male | 267 (3.47) | 485 (1.58) | 2.16* (1.86, 2.52) |
|  |  | All | 635 (4.23) | 1188 (1.98) | 2.10* (1.90, 2.32) |

RR, relative risk

* indicates the statistical significance after the Bonferroni correction.

sTable 4. Relative Risk of ASD between FDRs of individuals who committed suicide and matched cohort, stratified by kinships

| Type of Affected Relative | Sex of relative | Sex of individual | Number of ASD (%) | | Adjusted RRǂ  (95C.I.) |
| --- | --- | --- | --- | --- | --- |
|  |  |  | FDRs of CS individuals | Matched Cohort |  |
| Any | Female | Female | 6 (0.06) | 32 (0.08) | 0.79 (0.33, 1.86) |
|  |  | Male | 47 (0.41) | 143 (0.31) | 1.30 (0.93, 1.82) |
|  |  | All | 53 (0.25) | 175 (0.20) | 1.21 (0.88, 1.65) |
|  | Male | Female | 22 (0.10) | 73 (0.08) | 1.20 (0.74, 1.93) |
|  |  | Male | 81 (0.33) | 311 (0.32) | 1.14 (0.89, 1.46) |
|  |  | All | 103 (0.22) | 384 (0.21) | 1.15 (0.92, 1.43) |
|  | All | Female | 28 (0.09) | 105 (0.08) | 1.08 (0.72, 1.64) |
|  |  | Male | 128 (0.36) | 454 (0.32) | 1.18 (0.97, 1.44) |
|  |  | All | 156 (0.23) | 559 (0.20) | 1.16 (0.97, 1.39) |
| Parents | Female  (mother) | Female | 5 (0.10) | 26 (0.12) | 0.77 (0.29, 2.01) |
|  |  | Male | 38 (0.56) | 121 (0.45) | 1.25 (0.86, 1.81) |
|  |  | All | 43 (0.36) | 147 (0.31) | 1.17 (0.83, 1.65) |
|  | Male  (father) | Female | 18 (0.15) | 65 (0.14) | 1.12 (0.67, 1.89) |
|  |  | Male | 71 (0.50) | 274 (0.48) | 1.13 (0.87, 1.46) |
|  |  | All | 89 (0.34) | 339 (0.33) | 1.13 (0.89, 1.42) |
|  | All | Female | 23 (0.13) | 91 (0.13) | 1.04 (0.66, 1.63) |
|  |  | Male | 109 (0.52) | 395 (0.47) | 1.16 (0.93, 1.43) |
|  |  | All | 132 (0.35) | 486 (0.32) | 1.13 (0.94, 1.38) |
| Offspring | Female  (daughter) | Female |  |  |  |
|  |  | Male |  |  |  |
|  |  | All |  |  |  |
|  | Male  (son) | Female |  |  |  |
|  |  | Male |  |  |  |
|  |  | All |  |  |  |
|  | All | Female |  |  |  |
|  |  | Male |  |  |  |
|  |  | All |  |  |  |
| Siblings | Female  (Sister) | Female |  |  |  |
|  |  | Male | 9 (0.34) | 22 (0.21) | 1.64 (0.75, 3.58) |
|  |  | All | 10 (0.20) | 28 (0.14) | 1.43 (0.69, 2.96) |
|  | Male  (brother) | Female | 4 (0.08) | 5 (0.03) | 3.20 (0.86, 11.94) |
|  |  | Male | 10 (0.20) | 37 (0.18) | 1.08 (0.54, 2.17) |
|  |  | All | 14 (0.14) | 42 (0.11) | 1.34 (0.73, 2.44) |
|  | All | Female | 5 (0.07) | 11 (0.04) | 1.82 (0.63, 5.24) |
|  |  | Male | 19 (0.25) | 59 (0.19) | 1.29 (0.77, 2.16) |
|  |  | All | 24 (0.16) | 70 (0.12) | 1.37 (0.86, 2.18) |

RR, relative risk

* indicates the statistical significance after the Bonferroni correction.

sTable 5. Relative Risk of ADHD between FDRs of individuals who committed suicide and matched cohort, stratified by kinships

| Type of Affected Relative | Sex of relative | Sex of individual | Number of ADHD (%) | | Adjusted RRǂ  (95C.I.) |
| --- | --- | --- | --- | --- | --- |
|  |  |  | FDRs of CS individuals | Matched Cohort |  |
| Any | Female | Female | 61 (0.61) | 202 (0.50) | 1.20 (0.90, 1.61) |
|  |  | Male | 270 (2.35) | 737 (1.60) | 1.50* (1.30, 1.74) |
|  |  | All | 331 (1.54) | 939 (1.09) | 1.43* (1.26, 1.63) |
|  | Male | Female | 148 (0.66) | 471 (0.52) | 1.26 (1.05, 1.52) |
|  |  | Male | 519 (2.14) | 1667 (1.72) | 1.31* (1.18, 1.45) |
|  |  | All | 667 (1.43) | 2138 (1.15) | 1.30* (1.19, 1.42) |
|  | All | Female | 209 (0.64) | 673 (0.52) | 1.24 (1.06, 1.46) |
|  |  | Male | 789 (2.21) | 2404 (1.68) | 1.37* (1.26, 1.49) |
|  |  | All | 998 (1.46) | 3077 (1.13) | 1.34* (1.24, 1.44) |
| Parents | Female  (mother) | Female | 58 (1.10) | 179 (0.85) | 1.28 (0.95, 1.73) |
|  |  | Male | 234 (3.48) | 614 (2.28) | 1.57* (1.34, 1.84) |
|  |  | All | 292 (2.43) | 793 (1.65) | 1.50* (1.31, 1.72) |
|  | Male  (father) | Female | 131 (1.10) | 417 (0.88) | 1.27 (1.04, 1.55) |
|  |  | Male | 466 (3.29) | 1517 (2.68) | 1.30* (1.16, 1.45) |
|  |  | All | 597 (2.29) | 1934 (1.86) | 1.29* (1.17, 1.42) |
|  | All | Female | 189 (1.10) | 596 (0.87) | 1.27 (1.07, 1.50) |
|  |  | Male | 700 (3.35) | 2131 (2.55) | 1.38* (1.26, 1.50) |
|  |  | All | 889 (2.34) | 2727 (1.79) | 1.35* (1.25, 1.46) |
| Offspring | Female  (daughter) | Female |  |  | 1.33 (0.14, 12.83) |
|  |  | Male |  |  | 1.33 (0.14, 12.82) |
|  |  | All |  |  | 0.89 (0.19, 4.11) |
|  | Male  (son) | Female |  |  | NA |
|  |  | Male |  |  | 0.67 (0.15, 2.98) |
|  |  | All |  |  | 1.00 (0.28, 3.54) |
|  | All | Female |  |  | NA |
|  |  | Male |  |  | 0.80 (0.23, 2.76) |
|  |  | All |  |  | 0.40 (0.09, 1.71) |
| Siblings | Female  (Sister) | Female | ≦3 (0.08) | 20 (0.20) | 1.17 (0.79, 1.74) |
|  |  | Male | 36 (1.37) | 123 (1.17) | 1.06 (0.73, 1.54) |
|  |  | All | 38 (0.75) | 143 (0.70) | 1.33 (0.74, 2.40) |
|  | Male  (brother) | Female | 15 (0.31) | 45 (0.23) | 1.44 (1.04, 1.98) |
|  |  | Male | 53 (1.05) | 147 (0.73) | 1.40 (1.06, 1.85) |
|  |  | All | 68 (0.68) | 192 (0.48) | 1.02 (0.59, 1.75) |
|  | All | Female | 17 (0.23) | 65 (0.22) | 1.33 (1.03, 1.70) |
|  |  | Male | 89 (1.16) | 270 (0.88) | 1.26 (1.01, 1.58) |
|  |  | All | 106 (0.71) | 335 (0.56) | 1.20 (0.90, 1.61) |

RR, relative risk

* indicates the statistical significance after the Bonferroni correction.

sTable 6. Relative Risk of Accidental death between FDRs of individuals who committed suicide and matched cohort, stratified by kinships

| Type of Affected Relative | Sex of relative | Sex of individual | Number of Accidental death (%) | | Adjusted RRǂ  (95C.I.) |
| --- | --- | --- | --- | --- | --- |
|  |  |  | FDRs of CS individuals | Matched Cohort |  |
| Any | Female | Female | 23 (0.23) | 46 (0.11) | 1.84 (1.11, 3.05) |
|  |  | Male | 87 (0.76) | 186 (0.40) | 1.73* (1.34, 2.24) |
|  |  | All | 110 (0.51) | 232 (0.27) | 1.75* (1.39, 2.21) |
|  | Male | Female | 63 (0.28) | 135 (0.15) | 1.64* (1.21, 2.22) |
|  |  | Male | 177 (0.73) | 405 (0.42) | 1.55* (1.30, 1.85) |
|  |  | All | 240 (0.51) | 540 (0.29) | 1.57* (1.35, 1.83) |
|  | All | Female | 86 (0.26) | 181 (0.14) | 1.68* (1.30, 2.18) |
|  |  | Male | 264 (0.74) | 591 (0.41) | 1.60* (1.38, 1.85) |
|  |  | All | 350 (0.51) | 772 (0.28) | 1.62* (1.43, 1.84) |
| Parents | Female  (mother) | Female | 9 (0.17) | 20 (0.10) | 1.60 (0.73, 3.49) |
|  |  | Male | 45 (0.67) | 82 (0.30) | 2.10* (1.46, 3.03) |
|  |  | All | 54 (0.45) | 102 (0.21) | 2.01* (1.44, 2.79) |
|  | Male  (father) | Female | 24 (0.20) | 40 (0.08) | 2.11 (1.26, 3.52) |
|  |  | Male | 79 (0.56) | 177 (0.31) | 1.59* (1.21, 2.07) |
|  |  | All | 103 (0.40) | 217 (0.21) | 1.68* (1.33, 2.13) |
|  | All | Female | 33 (0.19) | 60 (0.09) | 1.93 (1.26, 2.97) |
|  |  | Male | 124 (0.59) | 259 (0.31) | 1.74* (1.41, 2.16) |
|  |  | All | 157 (0.41) | 319 (0.21) | 1.78* (1.47, 2.16) |
| Offspring | Female  (daughter) | Female | 12 (0.51) | 13 (0.14) | 3.44 (1.54, 7.66) |
|  |  | Male | 17 (0.79) | 68 (0.79) | 0.91 (0.53, 1.56) |
|  |  | All | 29 (0.65) | 81 (0.45) | 1.32 (0.86, 2.03) |
|  | Male  (son) | Female | 32 (0.56) | 77 (0.34) | 1.44 (0.95, 2.18) |
|  |  | Male | 60 (1.20) | 151 (0.76) | 1.47 (1.09, 1.97) |
|  |  | All | 92 (0.86) | 228 (0.53) | 1.46 (1.15, 1.86) |
|  | All | Female | 44 (0.55) | 90 (0.28) | 1.72 (1.20, 2.48) |
|  |  | Male | 77 (1.08) | 219 (0.77) | 1.29 (0.99, 1.67) |
|  |  | All | 121 (0.80) | 309 (0.51) | 1.42* (1.15, 1.75) |
| Siblings | Female  (Sister) | Female | 2 (0.08) | 13 (0.13) | 0.63 (0.14, 2.80) |
|  |  | Male | 25 (0.95) | 36 (0.34) | 2.57* (1.53, 4.32) |
|  |  | All | 27 (0.53) | 49 (0.24) | 2.06 (1.28, 3.31) |
|  | Male  (brother) | Female | 7 (0.14) | 18 (0.09) | 1.56 (0.65, 3.73) |
|  |  | Male | 38 (0.75) | 77 (0.38) | 1.68 (1.15, 2.47) |
|  |  | All | 45 (0.45) | 95 (0.24) | 1.64 (1.16, 2.34) |
|  | All | Female | 9 (0.12) | 31 (0.11) | 1.20 (0.57, 2.53) |
|  |  | Male | 63 (0.82) | 113 (0.37) | 1.93* (1.42, 2.62) |
|  |  | All | 72 (0.48) | 144 (0.24) | 1.76* (1.33, 2.33) |

RR, relative risk

* indicates the statistical significance after the Bonferroni correction.

sTable 7. Relative Risk of suicidal death between FDRs of individuals who committed suicide and matched cohort, stratified by kinships

| Type of Affected Relative | Sex of relative | Sex of individual | Number of suicidal death (%) | | Adjusted RRǂ  (95C.I.) |
| --- | --- | --- | --- | --- | --- |
|  |  |  | FDRs of CS individuals | Matched Cohort |  |
| Any | Female | Female | 74 (0.74) | 37 (0.09) | 7.38* (4.92, 11.07) |
|  |  | Male | 104 (0.90) | 65 (0.14) | 5.99* (4.38, 8.17) |
|  |  | All | 178 (0.83) | 102 (0.12) | 6.50* (5.08, 8.31) |
|  | Male | Female | 104 (0.46) | 87 (0.10) | 4.21* (3.17, 5.60) |
|  |  | Male | 196 (0.81) | 187 (0.19) | 3.77* (3.08, 4.63) |
|  |  | All | 300 (0.64) | 274 (0.15) | 3.91* (3.32, 4.62) |
|  | All | Female | 178 (0.55) | 124 (0.10) | 5.81* (4.62, 7.31) |
|  |  | Male | 300 (0.84) | 252 (0.18) | 4.35* (3.67, 5.16) |
|  |  | All | 478 (0.70) | 376 (0.14) | 4.61* (4.02, 5.29) |
| Parents | Female  (mother) | Female | 30 (0.57) | 16 (0.08) | 7.02* (3.76, 13.12) |
|  |  | Male | 48 (0.71) | 18 (0.07) | 10.46* (6.08, 18.01) |
|  |  | All | 78 (0.65) | 34 (0.07) | 8.74* (5.82, 13.12) |
|  | Male  (father) | Female | 33 (0.28) | 35 (0.07) | 3.83* (2.39, 6.13) |
|  |  | Male | 79 (0.56) | 66 (0.12) | 4.41* (3.18, 6.13) |
|  |  | All | 112 (0.43) | 101 (0.10) | 4.06* (3.10, 5.31) |
|  | All | Female | 63 (0.37) | 51 (0.07) | 4.51* (3.11, 6.53) |
|  |  | Male | 127 (0.61) | 84 (0.10) | 5.66* (4.30, 7.46) |
|  |  | All | 190 (0.50) | 135 (0.09) | 5.22* (4.18, 6.51) |
| Offspring | Female  (daughter) | Female | 30 (1.28) | 12 (0.13) | 10.33* (5.22, 20.43) |
|  |  | Male | 33 (1.54) | 28 (0.33) | 4.36* (2.61, 7.28) |
|  |  | All | 63 (1.41) | 40 (0.22) | 5.74* (3.84, 8.58) |
|  | Male  (son) | Female | 48 (0.84) | 30 (0.13) | 5.65* (3.58, 8.91) |
|  |  | Male | 79 (1.58) | 77 (0.39) | 3.73* (2.70, 5.15) |
|  |  | All | 127 (1.19) | 107 (0.25) | 4.27* (3.28, 5.55) |
|  | All | Female | 78 (0.97) | 42 (0.13) | 6.52* (4.47, 9.50) |
|  |  | Male | 112 (1.57) | 105 (0.37) | 3.88* (2.95, 5.10) |
|  |  | All | 190 (1.25) | 147 (0.24) | 4.65* (3.74, 5.80) |
| Siblings | Female  (Sister) | Female | 14 (0.57) | 9 (0.09) | 6.36* (2.75, 14.71) |
|  |  | Male | 23 (0.87) | 19 (0.18) | 4.46* (2.41, 8.26) |
|  |  | All | 37 (0.73) | 28 (0.14) | 4.97* (3.01, 8.19) |
|  | Male  (brother) | Female | 23 (0.47) | 22 (0.11) | 3.82* (2.12, 6.90) |
|  |  | Male | 38 (0.75) | 44 (0.22) | 3.06* (1.97, 4.75) |
|  |  | All | 61 (0.61) | 66 (0.17) | 3.30* (2.32, 4.70) |
|  | All | Female | 37 (0.50) | 31 (0.11) | 4.45* (2.75, 7.21) |
|  |  | Male | 61 (0.79) | 63 (0.20) | 3.46* (2.42, 4.93) |
|  |  | All | 98 (0.65) | 94 (0.16) | 3.78* (2.84, 5.03) |

RR, relative risk

* indicates the statistical significance after the Bonferroni correction.
